# The impact of COVID-19 on Medical education and Medical Students. How and when can they return to placements?

**DOI:** 10.15694/mep.2020.000159.1

**Published:** 2020-08-05

**Authors:** Colin Macdougall, Peter Dangerfield, David Katz, W David Strain

**Affiliations:** 1 Warwick Medical School; 2 School of Medicine; 3 Division of Infection and Immunity; 4 Diabetes and Vascular Medicine Research

**Keywords:** Covid-19, Clinical teaching, Placement learning, Clerkships, Medical student, Health and Safety

## Abstract

This article was migrated. The article was marked as recommended.

The defining feature of 2020 will be the early and mid-stages of the covid-19 pandemic, declared by the World Health Organisation on 11 ^th^ March. Rapid worldwide exponential spread continues and by 15 April, more than 1 900 000 cases and 123 000 deaths had been reported worldwide (WHO, 2020).

Health services have coped to varying degrees. One common feature has been the withdrawal of routine care (Iacobucci, 2020a) and ‘non-essential’ staff including learners, although many have returned to undertake care roles. As the likely timeframe for stabilisation of health services becomes clearer, certainly in the United Kingdom (UK) (Iacobucci, 2020b), medical educators need to rapidly get the teaching of the next generation of health care workers back on track if they are to enter health services as confident and competent practitioners in 2020 and 2021.

Although a ‘whole world’ experience, the effects of covid-19 sit in national contexts. We detail the issues for the UK in re-starting and re-inventing medical education, noting that the principles, if not necessarily the detail, will be common across the world.

## Introduction

In a healthcare crisis, it is appropriate for the initial focus to be on the immediate life-saving actions. However, in parallel, ‘collateral damage’ must be avoided. To achieve this, substantial potential downstream problems including impact on care for unrelated conditions, needs considered ( Godlee, 2020).

In the current Covid-19 crisis UK Medical Schools moved quickly to bring senior students into the workforce (either paid or as volunteers) including many schools graduating their final year students early in order to gain provisional registration ahead of their due date in August 2020. These students entered the workforce to support clinical services from April onwards ( Patel *et al.*, 2020). Furthermore, Medical Academics were requested to provide direct care and the impact of this on UK academic medical research is likely to be very significant ( Banerjee *et al.*, 2020).

UK Health Care Education now needs to be a key national focus. In March 2020, as a response to the pandemic, standard clinical placements for medical students in earlier stages of their courses were withdrawn due to a combination of loss of routine services, redeployment of specialist staff and health concerns for and from learners on placement. The resultant gap in placement learning can be substituted for some students by volunteering but this is neither suitable for all nor likely to cover the range of clinical experience required to complete a UK primary medical qualification (PMQ). Therefore there is an urgent need to plan how, and critically when, medical students will return to placements that offer the necessary support to ensure successful and safe completion of their courses. Without this, the availability, competence and confidence of doctors entering the workforce in 2021 and beyond is in jeopardy.

At a time of huge uncertainty, with reduced availability of academic and clinical staff who are currently working alongside their colleagues in crisis mode ( RCP, 2020) there is currently no clear blueprint and timeline for return to something equating to ‘Medical School normality’. In addition, the crisis has itself driven changes in clinical practice which may change the future for which we are training these students.

For this to happen medical educators will need to work with and have support from Governments and professional regulators in the coming months and years. All stakeholders need to understand and acknowledge the current contribution and achievements of health care and health care education during this crisis but also facilitate schools dealing with the many challenges ahead. Of key importance is that the re-launching, adapting and supporting of high standards of clinical placements is crucial and has to be integral to the very early stages of restoration of health care.

## The immediate challenges

As noted, many Medical Schools took urgent action to graduate final year students, often earlier than usual. Ideally, Medical Schools should retain a role in mentorship, guidance and transition until the normal planned end of the course but this is not formally mandated after graduation, and some schools choose to offer pre-graduation work versions of assistantships ( GMC, 2015) instead of keeping formal mechanisms in place ( Patel *et al.*, 2020).

The majority of these students had already finished mandatory rotations and were entering a transition period of preparation for practice, e.g. assistantship, and/or work shadowing ( GMC, 2015). Such programmes were mainly transformed into supporting roles, in the context of limiting “risk” of direct interaction with Covid-19 patients. A considerable number of these students (possibly > 60%) started working on band 3 or 4 in the NHS ( Burr, 2009).

Students in the early years of courses, including even vertically integrated courses ( de Cates, Owen and Macdougall, 2018), have less direct clinical time and more teaching that can be replaced by distance and/or e-learning. For example, lectures, seminars and group work can be arranged at a distance using appropriate software, although personal circumstances influence how effectively an individual may be able to engage. For early year students, there is also time to adjust the remainder of the course to ensure competencies are achieved.

For more senior clinical students, however, delays in clinical rotations and availability of placements will have a significant impact on the ability to attain proficiencies. In particular, medical students in the penultimate year are required to be exposed to critical learning involving patient contact in a variety of care settings. They have limited time to make up for lost opportunities. Courses with early final examinations may be particularly challenged.

The potential ‘latest date’ of return to placements depends on curricula and course structure, but even a full September restart (which may not be possible for all) would equate to around 6 months of lost experience. Further, four year graduate entry programmes have less time overall and have a higher proportion of clinical exposure than more traditional courses ( de Cates, Owen and Macdougall, 2018). If August 2021 is to remain the start point for entering work (foundation programmes ( Raine *et al.*, 2018)) for those in their penultimate year, these issues need to be addressed immediately. Unless effective placements are resumed by September 2020 at the latest, there is a significant risk of delay in course completion and graduation in 2021.

A compounding problem is that a full range of services may not be back to ‘business as usual’ for some time. In addition, some services that used to take students might either no longer be available or may have reconfigured care such that the experience is very different. Shifts in outpatient, general practice and psychiatric care to tele-medical services will require re-visiting the learning available and whether this matches current or future need. Medical Schools cannot rely passively for services to be ready. The current Covid-19 predictions include a range of scenarios based upon major societal impact for several years ( Kissler *et al.*, 2020). It is obvious that NHS services cannot remain entirely Covid-19 focused for that long, so restoration of routine and non-urgent aspects of care is already being initiated. It is essential that Medical Schools and Medical Educational needs are taken into account in that planning.

## What is needed for return of students to satisfactory clinical placements?

Return to effective placement learning needs a sufficient range of placements in appropriate settings with sufficient capacity to support learners and keep them safe. There are several different necessary components:

### i Learning and Teaching

It is clear as of May 2020, that standard clinical placements will not be viable for some time. If medical schools re-open between July and September, this leaves limited time to complete 13-14 months of planned experience before July 2021 graduation and August 2021 entry into foundation programmes. The Medical Schools themselves, placement providers, regulators and Government need to focus on maximising opportunities in placements, bearing in mind the loss of experience taking place at present and explore the scope for moving some outcomes into postgraduate learning.

For placements to be beneficial, there will need to be an appropriate balance of clinical activity that matches the areas that the particular student cohorts need. Patient flows need to be sufficient and patients comfortable with interaction with learners. For example, obstetric care continues, but with significant limitation on the presence of partners. Would learners be seen as acceptable to patients if their own partners are being denied access?

Staff will need time to prepare new material that is flexibly enough to be delivered electronically if necessary should further surges occur, as well as to teach and support students directly. Currently many services are quieter than usual allowing more staff focus on teaching, but this is unpredictable and as practice returns to normal there will be significant “catch-up” increasing clinical demand. Medical students on placement will need to be safe and their Universities will have to recognise that this role and the contrast in risk profile with non-clinical learners.

Attendance for an individual learner is not itself critical to service but the inability to graduate large numbers of competent doctors will impact substantially on future care. Appropriate Personal and Protective Equipment (PPE), access to testing if unwell, ability to self-isolate and vaccination (if and when available) are required. The majority are not in high risk groups, but some students have background health issues, pregnancy etc. that would limit their ability to fully engage. Medical schools must be prepared for students needing intensive care and the possibility of student deaths.

### ii Clinical assessments

Demonstration of clinical capabilities is critical to patient safety and public confidence in medical care. Challenges in assessing clinical students during Covid-19 are obvious as students, patients (real or actors) and examiners are exposed to risk, especially where candidates have a sequence of brief contacts with a large number of individuals such as during OSCEs ( Harden, 2016).

More generally, it is naïve to assume that effective assessment ensures patient safety. Indeed, the GMC note it is only part of one necessary theme along with environment, culture, support and governance ( GMC, 2015), Importantly, it is what we do, not what we test, that ensures competence and competence: “weighing a pig doesn’t make it heavier” (C. F. Macdougall, 2010). This runs contrary to the 21 ^st^ century UK zeitgeist, with high stakes end point assessments being seen as the main or only route to maintain standards. The irony being that an early UK government action in the crisis was to cancel national school exams (In England, GCSEs and A levels), shifting to use of in course data ( Ofqual, 2020), despite having argued for years that this was not an appropriate way of assessing. Within medicine, this has manifested by the contrast between the planned launch of a national licensing exam (UKMLA) and the class of 2020, with many students graduating with a much reduced and changed examination diet and reliance on in course assessments.

For 2020/21 it is clear that Medical Schools will need to look at when and how students are examined to ensure student, faculty and patient safety from infection, whilst still being confident that the tests are robust. Some schools implemented a radical approach of open book online exams ( Ali, 2020), and remote proctoring, although such mechanisms were already in-train for the Foundation Allocation process ( UKFPO, 2020). Whatever the option chosen, the process of preparing, quality assuring and potentially accrediting individual Medical School system(s) needs to be put in place rapidly.

### iii Interim measures

There are several interim measures needed to ensure that medical education continues to maintain high standards. There is no specified length for placements but it will be necessary to shorten them. This has to be considered against a background of many courses already struggling to find sufficient time for some experiences and further shortening at a time of extreme service pressure is not opportune. There is also a balance between an earlier re-start into a less stable care setting versus waiting without a guaranteed of future stability, noting that further substantial disruption including further periods of lockdown are likely ( Kissler *et al.*, 2020).

Prior to return to placements, students could undertake non-patient facing activities usually undertaken alongside practice. Introductory and supportive lectures and seminars, skills teaching, teaching about clinically applicable laboratory and imaging, appropriate prescribing tasks and group work such as problem-based learning could all be brought forward in order to maximise time when at the clinical placement coalface. If this happens, it is vital that the impact of the decoupling teaching and reflection on practice is recognised with classical experiential learning cycles being lost ( Kolb, 1984). Such changes therefore have to be carefully prepared and structured.

Once placements restart, a clear focus will be ensuring quality learning is achieved in less time. Clinical learners are often passive observers which is a low yield use of an increasingly valuable resource. Learners will need supportive teachers with time to teach and ‘just in time’ supportive learning from resources online and via apps. Every learning opportunity needs to be taken, even in busy environments, although models have existed for a long time ( Hargreaves, 1997). Again, new learning environmental structures will need to be created to support this.

Simulation is often used to support clinical learning, particularly around medical procedures and high-risk situations such as emergency care as a key adjunct to improving patient safety, although this is highly staffing dependant ( Acton *et al.*, 2015). Increased use of simulation, for example for consultation skills and routine examinations, could be done with better social distancing and improved infection control than in the relatively uncontrolled clinical environment. This often needs funding separately from standard placement tariff and funding agencies across the UK (HEE, NHS Education For Scotland, HEIW and the NI MDTA) need to address this need, although some capacity may come from public volunteering.

There are other key areas with particular financial issues that need to be taken into account. Community care is being re-structured and the Covid-19 crisis has highlighted how provision of care in care homes and patients’ homes has a different risk profile to acute hospital trusts. Some services are only marginally staffed in normal times and there are complex funding issues, with allocation of National Undergraduate Tariff (NUT) to cover education varying nationally. Such funding issues apply particularly to General Practice. Psychiatric care also shares historic funding and staffing challenges.

### iv. Timing options

In order to ensure as much clinical placement time as possible Medical Schools will have to reduce, drop or adapt activities.

Learners were often focused at particular times of the day / week, only undertaking ‘out of hours’ learning appropriate to the topic, such as in emergency care. NHS restoration plans will likely include increased evening and weekend work. Medical Schools need to exploit learning opportunities across time, while ensuring appropriate allowance is made for those with health, caring or other needs. E-rostering systems could help to ensure opportunities are made available to learners.

Student selected components including electives and in particular the classical overseas elective (C Macdougall, 2006a) may not be possible due to travel restrictions. Most were cancelled in 2020. Whereas this time has been viewed as an opportunity to make up lost experiences, electives often provide valuable clinical experience and confidence (C Macdougall, 2006b). Additionally, these flexible course elements allow some students time to remediate and/or resit assessments.

The timeline of moving into work could also be re-visited, with assistantship elements ( GMC, 2015) moving until after graduation to become a lengthened shadowing period, noting that the objectives of these two remain distinct. Also, this means loss of the academic oversight by schools, although we would hope new graduates would take the transition very seriously. More radically, schools and the foundation programme could consider the final year and first foundation year as a more direct continuum, working collaboratively to re-define the pre- and post-graduation requirements and move some requirements later into Foundation training. This would need a regulatory plan.

#### Regulation

The UK regulator, the GMC, stipulate outcomes that focus appropriately on patient safety. The GMC were very clear in March 2020 that the standards were unchanged and that the responsibility to assess this lay with the Universities, and thus the Medical Schools;

“We have confirmed that it is a university’s decision when to graduate a student. But you can submit your graduation list sooner than normal, as long as your students:

• Have satisfied our professional requirements
• Met our curricula outcomes to make sure they have the skills and knowledge, to give patients the best possible care as new doctors" ( Amison, 2020).

Whilst this stance is superficially attractive, it may not be sustainable. In 2020, graduates lost up to three months placements time after a largely undisrupted course. As such, it could be argued that they had already achieved necessary outcomes. Today’s penultimate year students due to graduate in summer 2021 will have missed several months of clinical attachments and will return to disrupted placements. They still require to be competent and confident to enter work and discussion is critical as to how and when they can achieve and demonstrate competence and whether this can be fully achieved by normal graduation times.

#### Student support

2020 is both an exceptionally exciting time to be studying or starting to study medicine but also a very concerning one. The importance of healthcare delivery and the staff that deliver is being celebrated in a way that has never been seen before, but the academic challenge of medical courses under lockdown are huge and the personal risk higher than at any point prior to the start of the antibiotic age. Indeed, there is a risk that we may be reverting to the period similar to when there were many descriptions of medical student morbidity and mortality from tuberculosis ( Brean and Kane, 1946).

At this time students will need a different sort of support delivered without face to face contact. Students in difficulty will be more isolated from faculty, peers and their personal and family support. They will be increasingly aware of infection risks and at raised risk of other health problem including mental health issues. Again, this needs to be recognised and resourced properly.

## Summary

Whilst daunting, none of these challenges will go away and are likely to be exacerbated if not confronted soon. We hope that all stakeholders will take a collaborative approach to curriculum, regulatory and educational requirements and standards.

## Take Home Messages

• All parties need to ensure that Medical Education is a core component of the NHS recovery plan nationally, regionally and locally with clear recognition of the critical timelines involved, including a commitment to a September 2020 return at the latest.
• Medical Schools must continue to work collaboratively to make optimum use of available learning resources, placements and teaching facilities.
• The GMC should recognise the need to look at standards for graduation in 2021 specifically and the potential requirement for variance.
• Health Education England and equivalents in the devolved nations must urgently identify funding for increased simulation and for increased use of online education compatible with both University and NHS sectors.
• Medical student support and guidance structures need to be adjusted to the new reality and made far more robust that they have been in the past.

## Notes On Contributors

The four authors are all elected members of the British Medical Association (BMA) Medical Academics Sub Committee (MASC).

Prof C Macdougall is Head of Medical Education at Warwick Medical School and is a clinical Paediatrician with an interest in Allergy. He has publications in medical educational topics and children’s allergy. ORCID: https://orcid.org/0000-0002-1452-9322

Prof Peter Dangerfield is based at the University of Liverpool and is involved in curriculum development, assessment and management. He has publications on PBL and use of WiKi in learning as well as extensive research into musculoskeletal problems in children.

Prof David Katz is Professor of Immunopathology, UCL; formerly Chair, Pathological Sciences Education Committee and Course Leader, Immunology/Cell Pathology Intercalated Programme; UCL MBPhD Committee; formerly chair of Fitness to Practice Panels, GMC/MPTS. Research interest in antigen presentation by dendritic cells in health and disease.

Dr W David Strain is Senior Clinical Lecturer at the University of Exeter Medical School with expertise in the microcirculation. His research focus is the health of older adults; ensuring the right patient gets the right treatment. Clinically, he runs a community diabetes service for the older adult. ORCID: https://orcid.org/0000-0002-6826-418X

## Declarations

The author has declared that there are no conflicts of interest.

## Ethics Statement

Ethical approval was not required for this personal view because it is not reporting research findings.

## External Funding

This article has not had any External Funding
