# Supplementary material for: The impact of COVID-19 on Medical education and Medical Students. How and when can they return to placements?
Source: MedEdPublish (2016). 2020 Aug 5;9:159. [Version 1] doi: 10.15694/mep.2020.000159.1 (PMC10702637; doi:10.15694/mep.2020.000159.1)
Supplement: Peer review history

# Reviewer report: Reviewer response for version 1

**DOI:** 10.21956/mep.20109.r31005

**Published:** 2020-08-27

**Authors:** Ronald M Harden

**Affiliations:** 1 AMEE

This review has been migrated. The reviewer awarded 4 stars out of 5 This is a useful read for all engaged in medical education at this difficult time. The article challenges us to think both of the short term and long term effect of the Covid pandemic on medical education. An interesting suggestion is that the pandemic may be a catalyst for a move to a closer continuum of education from undergraduate through postgraduate education.The effect on learning outcomes is noted but perhaps more attention could have been paid to competency based education and adaptive learning.The article reflects the changes in medical education identified in the recent BEME rapid review to be publishes in Medical Teacher

Reviewer Expertise:

NA

No decision status is available

# Reviewer report: Reviewer response for version 1

**DOI:** 10.21956/mep.20109.r31004

**Published:** 2020-08-08

**Authors:** Balakrishnan Nair

**Affiliations:** 1 Centre for Medical Professional Development and University of Newcastle

This review has been migrated. The reviewer awarded 4 stars out of 5 The COVID pandemic had been challenging for health care systems across the world . Medical schools had the problem of preparing the workforce for the future in these difficult times, when clinical placements had been challenging . So this article is timely and looks at the problems and potential solutions . Most schools have utilised this opportunity to innovate, and problem solve . Even though the article is UK centric , there are lessons for the global community . Most students have reported that these opportunities, while challenging , gave them the platform to step up their performance and becomes professionals sooner than anticipated. Students also learn that providing health care , at times, can be risky. There may be legal issues also I think the article could have expanded on the need for student support and some evaluation for the need to further assessments after graduation , if needed.

Reviewer Expertise:

NA

No decision status is available

# Reviewer report: Reviewer response for version 1

**DOI:** 10.21956/mep.20109.r31003

**Published:** 2020-08-08

**Authors:** Megan Anakin

**Affiliations:** 1 University of Otago

This review has been migrated. The reviewer awarded 4 stars out of 5 Thank you for writing this personal view/opinion piece that documents your experience of COVID-19 on medical students and their programmes in the UK. We encountered similar issues in New Zealand during our country-wide lockdown from March to May and our medical school is still addressing the gaps in clinical experiences for all of our medical students. The issues raised by a prolonged programme disruption are addressed with thoughtful consideration of learning experiences that will help medical students learn safely. This article will be of interest to educators in other regions of the world whose programmes and students have been similarly disrupted by the COVID-19 pandemic.

Reviewer Expertise:

NA

No decision status is available

# Reviewer report: Reviewer response for version 1

**DOI:** 10.21956/mep.20109.r31006

**Published:** 2020-08-07

**Authors:** Subha Ramani

**Affiliations:** 1 Harvard Medical School, Brigham and Women's Hospital

This review has been migrated. The reviewer awarded 4 stars out of 5 The pandemic has indeed affected all aspects of life around the world and as the authors state the world of medical education has been impacted in similar and different ways than other professional education. The difference is what medical students are being trained for and their contribution to the care of patients.This is a well written article on how the pandemic has impacted medical student education, in fact disrupted it. I completely agree with the authors that recovery should include plans to reorganize the return of medical students to their training, especially clinical training. Non-clinical education can be provided virtually but there is no substitute for real-patient exposure on the clinical side.The authors have described very well a set of key steps that national bodies, hospitals and educational leaders need to address before returning students safely back to the clinical education space. Though they have focused on the United Kingdom, many of these guidelines could be applicable elsewhere. The article does mention student support, I would also think about engaging students in co-creating the educational plan as there is nothing like getting their input into their perceived needs and feeling of safety.The article will be useful to all educational leaders and institutions that provide clinical placements for medical students and other health professions students, though steps enacted by regulatory bodies will differ from region to region.

Reviewer Expertise:

NA

No decision status is available
